# Management of patients with issues related to opioid safety, efficacy and/or misuse: a case series from an integrated, interdisciplinary clinic

**DOI:** 10.1186/s13722-016-0050-0

**Published:** 2016-01-28

**Authors:** William C. Becker, Jessica S. Merlin, Ajay Manhapra, Ellen L. Edens

**Affiliations:** VA Connecticut Healthcare System, 950 Campbell Avenue, Mail Stop 151B, West Haven, CT 06516 USA; Yale University School of Medicine, 367 Cedar Street, New Haven, CT 06510 USA; University of Alabama at Birmingham, BBRB 220a, 1720 2nd Avenue South, Birmingham, AL 35233 USA

**Keywords:** Chronic pain, Opioid use disorder, Prescription drug misuse, Opioid safety, Interdisciplinary care, Integrated care

## Abstract

**Background:**

Patients, providers, communities and health systems have struggled to achieve balance between access to opioid treatment for chronic pain and its potential harmful consequences: especially misuse, addiction and overdose. We developed an interdisciplinary clinic embedded within primary care (the Opioid Reassessment Clinic—ORC) with the goal of improving the quality of care of patients with co-occurring chronic pain and issues related to opioid safety, efficacy and/or misuse.

**Case descriptions:**

We present three cases referred to the ORC that highlight complex clinical scenarios related to assessment and treatment of patients with chronic pain and issues related to opioid safety, efficacy and misuse.

**Discussion and evaluation:**

In the context of the three cases, with respect to assessment, we discuss: making the diagnosis of opioid use disorder; allowing the patient space to endorse lack of efficacy; identification of co-occurring hazardous alcohol use; and recognizing barriers to multimodal pain care. With respect to treatment, we discuss: making a change in treatment with which the patient may not agree; effectiveness of buprenorphine/naloxone for the treatment of chronic pain; responding to low efficacy; and making continued opioid therapy contingent on engagement with substance abuse treatment.

**Conclusions:**

The core components of our approach—biopsychosocial assessment and multimodal treatment planning with an emphasis on promoting functional goals and safety using clear communication and a patient-centered stance—should guide providers in the management of similar clinical scenarios. More evidence is needed to definitively guide specific interventions and points of clinical equipoise.

## Background

While rates of opioid prescribing for pain may have reached a plateau in the US, adverse outcomes related to opioid therapy, most notably unintentional overdose deaths, remain a serious public health problem [1]. Patients, providers, communities and health systems have struggled to achieve balance between access to opioid treatment for chronic pain and potential harmful consequences of long term opioid therapy, especially misuse, addiction and overdose. Regulatory agencies and expert groups have published prescriber guidelines aimed at improving uptake of evidence-based practices including: (1) use of opioid treatment agreements; (2) regular monitoring for efficacy, safety and misuse using tools such urine drug testing and querying prescription monitoring databases; and (3) provision of or referral to addiction treatment if recurrent misuse or opioid use disorder is identified [2, 3]. These are becoming the standard of care for individuals on long-term opioid therapy.

In practice, primary care providers (PCPs), responsible for most of chronic pain management and opioid prescribing in the US, struggle to follow these guidelines, citing lack of clinic time for opioid management, inadequate training, and low confidence [4]. Use of opioid risk mitigation strategies has been low in primary care [5], even among patients at high risk for misuse [6]. It is therefore understandable that the most complex cases—those in which individuals on long-term opioids, often at high doses, have persistently poor function, exhibit concerning behaviors such as running out of opioids early, or develop a substance use disorder to an opioid or other drug—are extremely challenging to manage in primary care. Notably, even among experts in the field, there is no consensus regarding how to best address these clinical situations.

In an effort to support primary care in the management of patients with complex chronic pain on long-term opioid therapy, a multi-disciplinary team designed the Opioid Reassessment Clinic (ORC) at VA Connecticut Healthcare System [7]. (Co-author JSM directs a chronic pain clinic for individuals with HIV, with a similar scope of practice as the ORC [8]).

Embedded within primary care and staffed by an addiction psychiatrist, an internist with addiction and pain training, a behavioral health advanced practice nurse (APRN) and a clinical health psychologist, the ORC has served as a learning opportunity for management of complex chronic pain and opioids over the past 3 years. The purpose of this study is to present actual ORC cases (with some details changed to preserve anonymity) as a platform to describe our approach to assessment and treatment, with the goal of highlighting evidence-based practices as well as some approaches we employ based on pragmatic considerations where evidence is lacking. While not intended to be a comprehensive clinical discussion of each case, our aim is to provide practicing clinicians guidance on generalizable issues related to pain, opioid safety and addiction within each case. We intend the discussion in each case to be relevant to generalists and specialists alike.

### ORC flow (see Fig. 1)

The team’s internist reviews all referrals and appropriate patients are scheduled for an initial intake. The team’s APRN performs a chart review prior to the initial evaluation covering information such as: summary of the consultation question, history of the pain complaint(s), engagement in multimodal pain treatment, current opioid dose and prescriber, evidence of aberrant medication-taking behavior, mental health diagnosis and degree of engagement in treatment, past medical history, and overall utilization of health services.

**Fig. 1:**
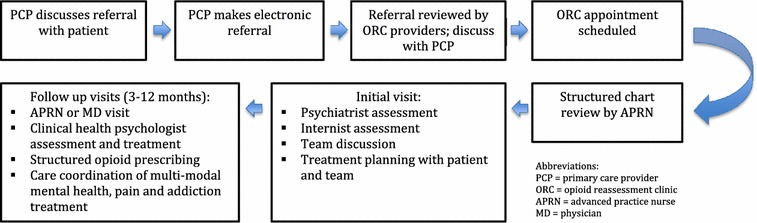
Opioid reassessment clinic flow

The patient is scheduled for a 1.5–2-h initial evaluation—the first part with the psychiatrist and subsequently, the internist. The psychiatric evaluation includes an assessment of pain generators, current level of functioning and pain interference, current treatment approaches, functional goals as identified by the patient, and the use and effectiveness of opioids for the pain condition. A discussion of any noted aberrancies occurs and education about current best-practice pain treatment is initiated. Additionally, a targeted mental health and substance use history is gathered along with any other pertinent medical history. Following this evaluation, the team meets to discuss the case and an initial plan is developed. The team internist then meets with the patient to discuss general impressions, perform a physical examination, elicit responses to any remaining questions, and collaborate with the patient in determining a plan.

An important point to emphasize in our general approach is the recognition that the intersection of chronic pain and opioid issues may be fraught with frustration, mistrust and dissatisfaction for both patients and providers. In view of this, we place special emphasis on transparency and clear communication both with the patient and teams of providers involved in the patient’s care. When communicating a potential treatment plan with the patient, we summarize our approach as the “7E’s”: **e**mpathize; **e**licit functional goals; **e**ducate; **e**ndorse an alternative plan consistent with patient’s functional goals; **e**nlist patient’s buy-in; **e**nact a follow-up plan; and **e**quanimity. Anecdotally, we find this approach to increase the likelihood of preserving a therapeutic alliance and the patient’s engagement in the treatment process. In case 1, we include how this approach was employed, including specific useful phrases.

**Table 1:** Core opioid reassessment clinic (ORC) treatment strategies in three cases

Case	Plan regarding opioid therapy	Plan regarding multi-modal pain treatment	Plan regarding management of co-occurring conditions
64-year-old with severe COPD, PTSD and degenerative joint disease of the lumbar spine, chronic pain and long-term, high-dose opioid therapy. Diagnosed with mild opioid use disorder (OUD) in ORC	Prior to OUD diagnosis: offered lower dose full-agonist opioid versus switch to buprenorphine/naloxone (BUP/NX) After OUD diagnosis: offered switch to BUP/NX maintenance or taper off When BUP/NX was deemed unsafe, it was discontinued and non-opioid pain treatment was continued	*Health psychology* met with patient at each ORC visit to establish functional goals and monitor progress *Pulmonary rehabilitation* engaged patient in upper body and breathing exercises	*Treatment of significant PTSD symptoms*. When a co-occurring condition is significantly contributing to pain, we make engagement in treatment of the condition part of the opioid treatment agreement
65-year-old with history of low back pain, cervical and lumbar surgeries, spinal cord stimulator placement and high-dose opioid therapy. No opioid misuse present. Assessed as having low efficacy, high-dose opioid therapy	Initially offered options: (1) taper down/off current opioid; (2) rotate to another full agonist, at a lower equivalent dose; (3) switch to BUP/NX for off-label treatment of pain. (Patient chose option 3) When BUP/NX was maximized without benefit, patient was offered a choice to taper off or switch to low dose full agonist opioid. He chose the latter option and is continued at a moderate opioid dose	*Health psychology* met with patient at each ORC visit for engagement in cognitive-behavioral therapy for pain *Non*-*opioid medications* were prescribed for neuropathic component of pain	Patient had no significant pain-impacting co-occurring conditions
56-year-old with alcohol use disorder and bilateral hip pain due to severe osteoarthritis treated with morphine. Exhibited opioid misuse and experienced a return to alcohol use	Initially, expectations regarding safe opioid use were made explicit; morphine was continued and closely monitored Following return to alcohol use, morphine was tapered off. Tramadol was continued as long as safe use is demonstrated	*Orthopedics* was engaged to develop a plan for hip replacement *Health Psychology* met with patient at each ORC visit for engagement in cognitive-behavioral therapy for pain *Non*-*opioid medications* were prescribed to treat inflammation	*Addiction treatment was a requirement for ongoing opioid therapy*. Breathalyzers were obtained at each visit along with urine drug testing. We collaborated closely with addiction treaters to ensure their ability to reinforce the pain care plan

### General ORC approach

Examples of typical reasons for referral to the ORC include: concerning medication-taking behaviors (e.g. request for an early refill or a urine drug screen with an unexpected result); sole reliance on opioids with limited or no engagement in other pain treatment modalities; unhealthy alcohol use; co-prescribed high-dose opioids and sedatives (most commonly benzodiazepines); significant co-occurring mental health symptoms; and an opioid use disorder on medication assisted therapy (i.e. methadone or buprenorphine/naloxone) with persistent, impairing pain. While these issues are complex, the principles we employ are the same standards for any patient with chronic pain, as described by the Institute of Medicine: biopsychosocial assessment and multi-modal pain treatment matched to the needs identified in the assessment [9]. As such, we structure each treatment plan around three core questions: What is the plan regarding opioid therapy?; What is the plan regarding multi-modal pain treatment?; and What is the plan regarding management of co-occurring conditions that will impact the likelihood of success of the pain care plan? This framework, including opioid management options described below, and how it was applied in the three cases is presented in Table 1.

### Emphasis on opioid safety

Regarding opioid therapy, typically three potential scenarios emerge from the initial assessment:

1. Misuse or safety without clear opioid use disorder: If misuse or safety are a concern, yet opioid use disorder is not clear, a trial of structured opioid therapy with or without an opioid dose reduction may be initiated. Structured opioid therapy consists of frequent monitoring of prescribed opioids as well as close follow-up of multimodal pain treatment engagement, achievement of functional goals, mood and patterns of medication use [10]. If after 3–9 months, patients are able to demonstrate safe medication use, improved function, and engagement in multimodal treatment, they are discharged back to primary care for continued implementation of the pain care plan. On the other hand, if addiction to opioids or other substances is identified during this period of structured monitoring, patients are transitioned to addiction treatment, including potentially initiation of buprenorphine/naloxone (BUP/NX) within ORC (both MDs are certified prescribers). If other safety issues emerge that do not require ongoing maintenance therapy, an opioid taper is initiated.
2. Clear lack of efficacy: If lack of efficacy is apparent–high levels of pain and limited or worsening function despite moderate or high dose opioids (≥50 mg morphine equivalent daily dose), without evidence of opioid misuse or addiction–opioid reduction is initiated, with the potential for complete discontinuation.
3. Clear opioid use disorder: If opioid use disorder is clear at the initial evaluation, a trial of medication assisted therapy (MAT) is provided either within our clinic (i.e. initiation of BUP/NX) or in a specialty SA treatment program.

### Case 1 presentation

This is a 64-year-old patient with severe chronic obstructive pulmonary disease, post-traumatic stress disorder (PTSD) and degenerative joint disease of the lumbar spine, accompanied by many years of chronic pain and long-term high dose opioid therapy. Three months before ORC referral, the patient was admitted with recurrent hypercapneic respiratory failure, confusion, and a fall with head trauma. The inpatient team counseled the patient that high-dose opioid therapy (in the patient’s case 240 mg morphine equivalent daily dose [MEDD], well above the most common definition of high-dose therapy: ≥100 mg) likely contributed to these events and that dose lowering or discontinuation would be necessary. The patient’s opioid dose was lowered on discharge; the patient’s significant dissatisfaction with this decision prompted the PCP to refer the patient to the ORC. Over several visits to ORC spanning several months, we carefully observed the patient’s behavior relative to opioids. The patient repeatedly focused the conversation on opioids to the exclusion of other treatment modalities, and was largely unwilling to engage in discussions about the harms of opioids to his health. Providers’ attempts at patient-centered discussions to this end were met with escalating anger and agitation by the patient. Additionally, despite setting clear expectations and boundaries regarding how to take his medications, he ran out early twice during this time. As a result, we made the diagnosis of mild opioid use disorder, elicited goals of care—to be more engaged with family and make more outings with them, and further assessed the patient as having marked deconditioning, poorly-controlled PTSD and non-engagement in evidence-based mental health and multi-modal pain treatments. We tapered the patient off of full agonist opioids, transitioning to BUP/NX at 4/1 mg TID and delivered mental health, addiction, and multi-modal pain treatments within our clinic. Thrice daily dosing of BUP/NX is a common practice when the drug is used for pain as it is believed to have a 6–8 h analgesic half-life [11].

### Case 1 discussion

#### Assessment: making the diagnosis of opioid use disorder

As described by many experts in the field [12–14], assessing the diagnostic criteria for opioid use disorder in a patient prescribed opioids for chronic pain can be challenging for a number of reasons. As per the DSM-5, tolerance (which this patient exhibited) and withdrawal symptoms cannot count towards the diagnosis in long-term opioid therapy for pain since some degree of tolerance is expected and withdrawal symptoms would likely occur if opioids were abruptly discontinued. Also, patients may characterize aberrant medication-taking behaviors (e.g. taking more opioid than prescribed) as pain-relief-seeking whereas providers may view these as evidence of loss of control [15]. To disentangle this, we rely heavily on the use of clear communication with the patient on what constitutes safe use, using a written treatment agreement to document the discussion. We clearly state: “If you are unable to follow these rules, you will have demonstrated that opioid treatment is not safe for you and we will stop it.” We view a patient’s inability to extinguish unsafe medication-taking behavior after he or she is aware that continued behavior will result in discontinuation of opioid therapy as, in and of itself, loss of control. Furthermore, recurrent behaviors inconsistent with the treatment agreement typically map onto DSM-5 criteria for opioid use disorder. For example, in this case, we made the diagnosis of mild opioid use disorder based on our assessment of the presence of three diagnostic criteria: craving, or a strong desire or urge to use opioids; recurrent opioid use in situations in which it was physically hazardous; and continued opioid use despite knowledge of having a persistent physical and psychological problems that were likely to have been caused or exacerbated by use.

#### Treatment: making a change in treatment plan with which the patient may not agree

In this case, the presence of opioid use disorder and life-threatening complications exacerbated by opioid therapy were two criteria for which ongoing opioid therapy were contraindicated. Prescribing full opioid agonists like oxycodone to someone with an opioid use disorder is explicitly discouraged by existing guidelines [16]. Despite this, providers in both the inpatient and outpatient settings continued full agonist opioid therapy for the treatment of pain; in our experience, this is not an uncommon occurrence. This paradox highlights the clinical challenges and uncertainties these situations present. We hypothesize that there are several reasons why physicians continue to prescribe full opioid agonists in individuals with opioid use disorders. First, as above, opioid use disorder can be challenging to diagnose especially in individuals on long-term opioid therapy for chronic pain. Second, clinical inertia—not making an indicated change in opioid regimen—may be partly driven by wanting to avoid conflict with the patient [17], who might have strong beliefs related to effectiveness and necessity of opioid therapy [18], and partly in general empathy towards the suffering of the patient [19].

Using the 7Es, we negotiated a switch to BUP/NX over several months. We expressed *empathy* by stating clearly, “We know your pain is real and can understand the thought of being in more pain is frightening and stressful.” We *elicited functional goals* by asking, “What would you like to be able to do that pain is keeping you from doing these days?” In this case, the patient noted that he was not interacting with family as he wished. He was provided *education* about the risks of high dose opioids emphasizing those risks most relevant to him (i.e. respiratory failure) and given a description of what safe opioid use would look like (not taking more than prescribed, only one pharmacy and provider, etc.) We *endorsed an alternative plan* of continuing the lowered opioid dosing with engagement of multimodal treatment. We pointed out that his functional goal (family engagement) wasn’t being met with the high doses of opioids. Though sometimes counterintuitive to patients, we repeatedly reinforced the concept that his function might actually improve on lower doses once he was less sedated and having fewer medical emergencies. Also, over time, his pain might improve as well with lower opioid doses and engagement in multimodal pain care. *Enlisting patient buy*-*in* was difficult with this patient given his singular focus on obtaining higher doses of opioids. Nonetheless, we worked to increase the patient’s buy-in by providing him with options for treatment when available and sharing decisions when possible. While the “macro” treatment decision in this case (namely, discontinuing full agonist opioids) was not a shared decision, we find that when there are “micro” decisions where flexibility is possible (for example, frequency of BUP/NX dosing), letting the patient’s preferences guide such decisions is an important way to build trust and buy-in. Additionally, motivational interviewing, with its emphases on eliciting a person’s innate motivation and linking stated priorities to positive behavior change, can be useful in securing patient buy-in. A follow up *plan was enacted* after discussion and agreement with the entire ORC team. In all of our interactions, we strive for *equanimity*. In this case, the patient was often upset with the team, believing he had been lied to about the possibility of going back on full agonist opioids, sometimes even screaming at providers. Having a team to process interactions and, when needed, to step in or provide follow up coverage has been helpful in maintaining calm and staying patient-centered in the face of patient emotions.

#### Treatment: effectiveness of BUP/NX for the treatment of chronic pain with co-occurring OUD

While a full review is beyond the scope of this study, there are a number of observational studies [20] and case series [21–23] suggesting that BUP/NX can be an effective treatment for chronic pain. As with many pain treatments, predicting who will respond to BUP/NX has proved more challenging [24]. An important generalizable point is that had the patient not tolerated BUP/NX, either because of poor efficacy or side effects, a return to full agonist opioids for the treatment of chronic pain would not have been offered because of the diagnoses of opioid use disorder and life-threatening opioid-related complications, unless the patient’s goals of care were to change to comfort measures.

#### Treatment: non-opioid options

A wide array of generally safer non-opioid pharmacologic and non-pharmacologic options exist for managing this patient’s chronic pain, some of which have superior efficacy data to support them. Along these lines, our message to all patients in the ORC is as follows: the data are clear that a multi-modal pain management approach works best in the long-run for helping our patients achieve their functional goals; let us work with you to identify which combination will work best for you.

Case 1 epilogue: After transitioning to BUP/NX, the patient had three more hospital admissions for COPD exacerbation with marked hypercapnia and somnolence. Dose reduction was employed; however, even with BUP/NX at 2 mg daily, markedly depressed sensorium was intermittently present. While non-adherence with home BiPAP and worsening COPD were perhaps most responsible for this symptom, we felt that BUP/NX was at best not helping the situation, and at worst, could be contributing. Additionally, the patient did not report improved pain with BUP/NX. Thus, it was discontinued altogether. The patient remains on non-pharmacologic and non-opioid pain treatments.

### Case 2 presentation

This is a 65-year-old patient with widespread degenerative joint disease and longstanding low back pain that has continued despite multiple cervical and lumbar surgeries and spinal cord stimulator placement. The PCP referred the patient to the ORC for consideration of an opioid rotation with dose reduction given tolerance to high doses of opioids (in this case, >200 mg MEDD). The patient was functionally quite active, pursuing an exercise-based hobby that required regular travel, and had no history of unsafe opioid use or misuse. However, the patient reported that the opioid regimen did not improve pain to a significant degree; he continued it because he had been on it for so long and worried what would happen if he were to stop. Therefore, in our clinical assessment, the patient did not have opioid use disorder, but would likely benefit—in terms of long-term safety—from decreasing the opioid dose. In the ORC, following a full assessment, the patient was provided a choice between (a) slow taper off opioids; (b) rotation to a different full agonist opioid but at a markedly lower dose; or (c) rotation to a trial of BUP/NX for treatment of chronic pain. After a thorough discussion, the patient favored the latter option given significant ambivalence about being on full agonist opioids long-term.

### Case 2 discussion

#### Assessment: allowing the patient space to endorse lack of efficacy

In our experience, some providers doubt that patients would endorse low or absent efficacy of long-term opioids, since doing so would threaten ongoing receipt of opioids. However, in observational studies, a substantial minority of patients self-discontinue opioids due to lack of efficacy [25]. As illustrated by this case and prior literature [26], patients understandably fear the potential for withdrawal symptoms that often occurs when opioids are discontinued abruptly. We have observed that if concerns about ongoing, high-dose opioid therapy are clearly explained, and patients are reassured that a taper would occur in a cautious, step-wise fashion with close oversight, patients seem open to disclosing lack of efficacy. Importantly, this patient discussed this with the PCP first, demonstrating that a specialist’s involvement was not needed upfront.

#### Treatment: responding to low efficacy

In our view, continuing ineffective opioid treatment is suboptimal, as it exposes the patient to cumulative opioid toxicity (e.g. osteoporosis, hypogonadism, and fall risk) with no corresponding benefit. In contrast to case 1, there was no evidence-guided indication for BUP/NX compared to rotation to a lower dose of a full agonist or taper off entirely, so the patient was given the choice. Also in contrast to case 1, had the trial of BUP/NX not succeeded, a return to full agonist opioids would have been considered, but, again, at a markedly lower dose. Of note, the use of BUP/NX in this setting is “off-label” insofar as it does not have FDA approval for treatment of chronic pain; however the DEA has endorsed such off-label use [27].

Case 2 epilogue: The patient made the transition to BUP/NX easily, taking his initial dose at home. After the first 2 weeks, his dose was increased from 12 mg daily (divided 4 mg TID) to 18 mg because of inadequately controlled pain. His dose was ultimately increased to 24 mg daily, yet he still experienced significant pain that was interfering with his function. The ORC team considered this a failed trial of BUP/NX and offered him the option to taper off BUP/NX with no opioid after versus rotation to lower dose oxycodone. The patient chose the latter option and is now on 10 mg oxycodone QID (60 mg MEDD), noting markedly improved pain control.

### Case 3 presentation

This is a 56-year-old patient with bilateral hip pain due to severe osteoarthritis that significantly interfered with functioning for which the patient was prescribed morphine for several years. Nine months prior to ORC referral, the patient successfully completed a residential treatment program for his long-standing alcohol use disorder while continuing on long-term opioid therapy. Presently, the PCP referred the patient to the ORC after two episodes of running out of opioid prescriptions early (without early refill) and a brief return to alcohol use. In the ORC, we facilitated engagement in outpatient alcohol treatment by making continued opioid therapy contingent upon adherence with follow-up and required frequent ORC visits including breathalyzer tests and urine drug screens. Following another return to drinking, the patient was admitted to an intensive outpatient alcohol use disorder treatment program and was simultaneously tapered off morphine, transitioning to tramadol with adjuvant non-opioid medications including an NSAID, gabapentin, and topical lidocaine. While tramadol, a weak mu-receptor agonist with more prominent serotonin and norepinephrine reuptake inhibition, was still a risky medication in this patient in recovery, it is classified by the DEA as less risky (schedule V compared to oxycodone and morphine’s schedule II designation); plus the patient’s tramadol dose was markedly lower in terms of mg MEDD.

Additionally, orthopedics was consulted and agreed to schedule hip replacement surgery contingent upon several months of documented alcohol abstinence. This motivated the patient to adhere to appointments and monitoring as required by both ORC and addiction treatment providers. While the patient continued to request a switch to a “stronger” opioid, the ORC repeatedly reiterated that this was not an option given the recent history of misuse, the lack of evidence of improved functioning on such medications, and benefit of being on the lowest possible opioid dose prior to surgery. After 6 months of abstinence from alcohol, safe use of tramadol, and engagement in multimodal therapy including ongoing SA treatment, the patient was discharged from ORC to follow-up with PCP while awaiting scheduled surgery.

### Case 3 discussion

#### Assessment: identification of co-occurring hazardous alcohol use

In this case, there was a documented history of alcohol use disorder, alerting us from the outset to the need for ongoing and frequent monitoring of alcohol use. In this patient, alcohol abstinence was the goal so we employed breathalyzer testing at each office visit and added on ethyl glucuronide (EtG) testing to each urine drug screen. As a highly sensitive assay [28], we find EtGs especially helpful in documenting abstinence but less helpful when some alcohol use is acceptable (e.g. in patients on low-dose opioid therapy without history of alcohol use disorder) and an easy test to incorporate into practice because the urine sample for the drug screen can be used. Relatedly, we believe alcohol abstinence should be a treatment goal for any patient on moderate- to high-dose of opioid therapy (≥50 mg MEDD) since NIAAA guidelines recommend abstinence in patients taking medications that enhance central sedation (http://www.niaaa.nih.gov/alcohol-health/overview-alcohol-consumption/moderate-binge-drinking).

#### Assessment: recognizing barriers to multimodal pain care

Not infrequently, patients are ambivalent about the benefits of multimodal care. In these instances, we employ education and motivational interviewing—components of the 7Es approach–in an attempt to help the patient recognize the discrepancy between their stated functional goals and their plans to reach those goals. On the other hand, there are instances where misunderstanding of addiction by healthcare providers can prove its own barrier [29]. It is very reasonable for medical and surgical specialists to expect that addiction be treated prior to any invasive procedure. However, lack of collaboration between specialists and PCPs often means delays for patients in getting appropriate and timely interventions.

#### Treatment: making continued opioid therapy contingent on engagement with addiction treatment

As stated above, opioid therapy should never be the sole treatment modality in chronic pain. We consider optimization of medical and psychiatric co-morbidities a necessary component of the multi-modal pain treatment approach. For example, attempts at optimization of diabetes control must accompany pain pharmacotherapy for the treatment of painful peripheral diabetic neuropathy. Analogously, we required engagement in addiction treatment as a pre-condition for ongoing opioid therapy in this patient, not only to mitigate risk, but to maximize potential benefit of the pain care plan. This requirement is communicated upfront with the patient as part of the treatment agreement.

Case 3 epilogue: The patient underwent total hip replacement and received 2 weeks of full agonist opioid treatment (MSIR 15 mg Q6 PRN) following the surgery. After the 2-week post-operative period, the patient was transitioned back to tramadol. He remains abstinent from alcohol and participates fully in his hip rehabilitation program.

## Conclusions

These three cases from the ORC were meant to represent common challenges at the intersection between chronic pain and opioid safety, efficacy and misuse. We summarized our approach related to several challenging issues regarding assessment and treatment. It is important to note that in this field where evidenced-based guidance is fairly sparse, the details of the treatment plans may vary from setting to setting. However, the core components–biopsychosocial assessment and multimodal treatment planning with an emphasis on promoting functional goals and safety using clear communication and a patient-centered stance—should hold constant.

Additionally, we recognize that the majority of pain care in the US is provided by front-line PCPs, providers who may not feel that the management strategies presented here fall within their scope of practice. Even among pain specialists, comfort with identifying and addressing opioid use disorders may vary. Chronic pain and addiction specialists like we have on the ORC team are helpful in such situations, but are not widely available. Until a systematic change occurs to increase the availability of such specialists, this disparity between need and availability will be present.

As the number of anecdotal recommendations above implied, there are several areas in this field where clinical research is needed: for example, comparative effectiveness of various opioid tapering and rotation strategies; randomized studies of BUP/NX for the treatment of chronic pain; and motivational interviewing techniques for these common scenarios. Of note, work is underway by an expert consensus group (including authors JSM and WCB) to help define best practices in providers’ management of unsafe medication-taking behaviors [30]. And, finally, as called for by the Institute of Medicine [9], improved pain-related instruction in undergraduate and graduate medical education programs is also a high priority in the path towards improved quality of care.

## References

[1] Centers for Disease Control and Prevention (2011). Policy impact: prescription painkiller overdoses.

[2] Chou R (2009). Clinical guidelines for the use of chronic opioid therapy in chronic noncancer pain. J Pain.

[3] The Management for Opioid Therapy for Chronic Pain Working Group (2010). VA/DoD clinical practice guideline for management of opioid therapy for chronic pain.

[4] Krebs EE (2014). Barriers to guideline-concordant opioid management in primary care—a qualitative study. J Pain.

[5] Becker WC (2011). Racial differences in primary care opioid risk reduction strategies. Ann Fam Med.

[6] Starrels JL, et al. Low use of opioid risk reduction strategies in primary care even for high risk patients with chronic pain. J Gen Intern Med. 2011;26(9):958–64. 10.1007/s11606-011-1648-2 PMC3157518 21347877

[7] Dorflinger LM (2014). Integrating interdisciplinary pain management into primary care: development and implementation of a Novel Clinical Program. Pain Med.

[8] Perry BA (2013). Characteristics of an ambulatory palliative care clinic for HIV-infected patients. J Palliat Med.

[9] Committee on Advancing Pain Research Care and Institute of Medicine (2011). Relieving pain in America: A blueprint for transforming prevention, care, education, and research.

[10] Kahan M (2011). Canadian guideline for safe and effective use of opioids for chronic noncancer pain Clinical summary for family physicians. Part 2: special populations. Can Fam Phys.

[11] Heit HA, Gourlay DL (2008). Buprenorphine: new tricks with an old molecule for pain management. Clin J Pain.

[12] Savage SR (2003). Definitions related to the medical use of opioids: evolution towards universal agreement [see comment]. J Pain Symptom Manag.

[13] Compton WM, Volkow ND (2006). Abuse of prescription drugs and the risk of addiction. Drug Alcohol Depend.

[14] Alford DP, Compton P, Samet JH (2006). Acute pain management for patients receiving maintenance methadone or buprenorphine therapy. Ann Intern Med.

[15] Upshur CC, Bacigalupe G, Luckmann R (2010). “They don’t want anything to do with you”: patient views of primary care management of chronic pain. Pain Med.

[16] Federation of State Medical Boards (2013). Model policy on the use of opioid analgesics in the treatment of chronic Pain.

[17] Nicolaidis C (2011). Police officer, deal-maker, or health care provider? Moving to a patient-centered framework for chronic opioid management. Pain Med.

[18] Schieffer BM (2005). Pain medication beliefs and medication misuse in chronic pain. J Pain.

[19] Lembke A (2012). Why doctors prescribe opioids to known opioid abusers. N Engl J Med.

[20] Becker WC (2015). Buprenorphine/naloxone dose and pain intensity among individuals initiating treatment for opioid use disorder. J Subst Abuse Treat.

[21] Malinoff HL, Barkin RL, Wilson G (2005). Sublingual buprenorphine is effective in the treatment of chronic pain syndrome. Am J Ther.

[22] Daitch J (2012). Conversion of chronic pain patients from full-opioid agonists to sublingual buprenorphine. Pain Phys.

[23] Roux P (2013). Buprenorphine/naloxone as a promising therapeutic option for opioid abusing patients with chronic pain: reduction of pain, opioid withdrawal symptoms, and abuse liability of oral oxycodone. Pain.

[24] Rosenblum A (2012). Sublingual buprenorphine/naloxone for chronic pain in at-risk patients: development and pilot test of a clinical protocol. J Opioid Manag.

[25] Noble M, et al. Long-term opioid management for chronic noncancer pain. Cochrane Database Syst Rev. 2010;1(1). 10.1002/14651858.CD006605.pub2 PMC6494200 20091598

[26] Weiss RD (2014). Reasons for opioid use among patients with dependence on prescription opioids: the role of chronic pain. J Subst Abuse Treat.

[27] Heit HA, Covington E, Good PM (2004). Dear DEA. Pain Med.

[28] Wurst FM (2003). Ethyl glucuronide discloses recent covert alcohol use not detected by standard testing in forensic psychiatric inpatients. Alcohol Clin Exp Res.

[29] Smith R (1999). Medicine and the marginalised: they deserve the best, not the poorest, care. Br Med J (BMJ).

[30] Merlin JS (2015). Common and challenging behaviors among patients taking long-term opioid therapy: Preliminary results of a Delphi study.

